# Polyphenolic Antioxidants in Bilberry Stems and Leaves: A Non-Targeted Analysis by Two-Dimensional NMR Spectroscopy and Liquid Chromatography–High-Resolution Mass Spectrometry

**DOI:** 10.3390/antiox13111409

**Published:** 2024-11-17

**Authors:** Anna V. Faleva, Nikolay V. Ulyanovskii, Alexandra A. Onuchina, Dmitry S. Kosyakov

**Affiliations:** Laboratory of Natural Compounds Chemistry and Bioanalytics, Core Facility Center “Arktika”, M.V. Lomonosov Northern (Arctic) Federal University, Northern Dvina Emb. 17, 163002 Arkhangelsk, Russia; n.ulyanovsky@narfu.ru (N.V.U.); a.onuchina@narfu.ru (A.A.O.); d.kosyakov@narfu.ru (D.S.K.)

**Keywords:** *Vaccinium*, secondary metabolites, extract, non-targeted screening, NMR, HPLC-HRMS

## Abstract

Compared with those of berries, the stems and leaves of the genus Vaccinium are important and underestimated sources of polyphenols with high antioxidant activity. In the course of this work, aqueous methanol extracts of the aerial parts of common bilberry (*Vaccinium myrtillus* L.) and bog bilberry *(Vaccinium uliginosum* L.) were studied to analyze the component compositions of their biologically active polyphenolic compounds. The aqueous methanol fractions of the stems and leaves of the studied samples contained 8.7 and 4.6% extractives, respectively, and were comparable in total polyphenol content, but presented significant differences in antioxidant activity. The identification of polyphenolic compounds was carried out via the following two-stage analytical procedure: (1) non-targeted screening of dominant structures via the 2D NMR method and (2) analysis of HPLC-HRMS data via the scanning of precursor ions for a specific ion. A total of 56 phenolic compounds were identified, including the glycosides quercetin, proanthocyanidins, and catechins, as well as various conjugates of caffeic and *p*-coumaric acids, including iridoids. Some of the latter, such as caffeoyl and *p*-coumaroyl hydroxydihydromonotropein, as well as a number of lignan glycosides, were described for the first time in *V. uliginósum* and *V. myrtillus*.

## 1. Introduction

Although the chemical composition of plant secondary metabolites and their impact on human health have been extensively researched, studies aimed at identifying new biologically active compounds in plant materials continue to be relevant [1]. Among them, phenolic acids and other polyphenolic compounds possessing a variety of biological activities (antioxidative, antidiabetic, antimicrobial, antiulcer, and others) are of great interest.

Being representatives of the genus *Vaccinium*, bog bilberry (*V. uliginósum*) and common bilberry (*V. myrtillus*) are characterized by a high content of polyphenolic compounds [2,3,4] and are widespread in temperate climate zones [3]. This led to the exceptional popularity of these plants in traditional folk medicine for the treatment of various conditions, such as stomatitis, fever and cough, diabetes, and infections of the digestive and urinary systems [5,6,7].

Kopystecka et al. [2] presented a detailed review of the metabolic profiles of both species and noted that *V. uliginosum* has not been extensively studied in terms of its metabolic composition compared to *V. myrtillus*. Moreover, most scientific research has focused on fruits (berries), which are commonly consumed in the human diet and are distinguished by a high content of anthocyanins [3,4,8,9,10]. Significantly fewer papers have described biologically active compounds found in leaves and stems [9,10,11,12], which are byproducts of berry harvesting and processing and have a noticeably higher content of polyphenolic compounds than fruits. Thus, the total phenolic content (TPC) of leaf extracts is reportedly ~16–20 mg/g dry weight [3], whereas for berries, this value falls within the range of 3.9–7.0. In light of this, it is not surprising that the antioxidant activity of *V. myrtillus* leaves is twice as high as that of fruits [13]. Flavonoids and phenolic acids, as well as their glycosylated derivatives, which are equally important for human health [14,15], form the basis of the chemical composition of polyphenols in the green parts of bilberry. The use of high-performance liquid chromatography with ion trap mass spectrometry detection allowed Bujor et al. [9] to tentatively (based on tandem mass spectra) identify about 100 compounds of these classes in *V. myrtillus* extracts (plant material was collected in Romania). Among them, caffeic acid derivatives (70–80% of total phenolics content) and flavanol oligomers (50–65%) predominated in leaf and stem extracts, respectively. Other detected compounds were assigned to the groups of coumaric acid derivatives, flavonol glycosides, and flavanol monomers.

A detailed comparative study of *V. uliginosum* and *V. myrtillus* harvested in Macedonia was conducted by Stanoeva et al. [3] using the same analytical methodology. They established the presence of 36 phenolic compounds belonging to 5 groups in the leaf extract: phenolic acids, flavonoids, catechin, and its derivatives, as well as iridoids and cinchonain. The main differences in the polyphenolic profiles of bog bilberry and common bilberry leaves were revealed in the chemical compositions of flavonols and flavan-3-ols. The presence of iridoid glycosides conjugated with cinnamic acids (mainly *p*-coumaric acid) is a distinctive feature of plants of the genus *Vaccinium* [16]. Iridoids have valuable pharmacological properties, including antitumor, neuroprotective, hepatoprotective, and anti-inflammatory effects [17].

Thus, despite the fact that bilberry has been well studied, the available data from the literature on the chemical composition of polyphenolic extractives in green parts of the plant are quite contradictory and cannot be considered exhaustive. To a large extent, this may be due to methodological problems associated with the difficulties of non-targeted search and identification of analytes in the extremely complex matrix of the plant extract.

Liquid chromatography–tandem mass spectrometry (HPLC-MS/MS) is a universal and powerful analytical technique in metabolomics [18] and has been repeatedly used in the analysis of the plant extracts of the genus *Vaccinium* [16,19,20,21,22]. However, various polyphenolic compounds may occur in both free and glycosylated forms or conjugate to each other, forming complex structures with higher molecular weights [23]. The resulting diversity of such analytes poses serious challenges in identifying and correctly assigning their signals in large arrays of mass spectrometric data. This problem can be partially solved by the use of high-resolution mass spectrometry (HRMS), which enables the selection of candidate compounds on the basis of their elemental compositions and greatly simplifies an interpretation of tandem mass spectra. For *Vaccinium* fruits, this approach was successfully used by Ancillotti et al. [21] and allowed for the reliable identification of more than 200 extractable compounds.

In our opinion, the HPLC-HRMS analysis of polyphenolic compounds in plant extracts may be greatly simplified by introducing complementary analytical techniques for structure elucidation and selection of appropriate candidate compounds. Among them, two-dimensional (2D) NMR spectroscopy, hyphenated with computational expert systems for data analysis and spectra prediction, has the greatest potential. Recently, this technique was successfully used in combination with HPLC-HRMS in the studies of secondary plant metabolites of cloudberry fruits and leaves [24], lignans of coniferous knotwood [25], and in the identification of a new class of compounds found in moss tissues [26]. The only example of this approach being applied to the analysis of bilberry secondary metabolites is a study by Liu et al. [27] in which the structures of three compounds (chlorogenic acid, arbutin, and 2-*O*-caffeoylarbutin), initially identified via HPLC-HRMS analysis, were confirmed by 2D NMR.

In the present study, aimed to provide deeper insight into the chemical composition of polyphenolic compounds in bog bilberry (*V. uliginósum*) and common bilberry (*V. myrtillus*) green parts, we propose an advanced two-stage 2D NMR/HPLC-HRMS analytical methodology. The latter involves the identification of principal polyphenolic structures in plant extracts by 2D NMR and an establishment on this basis of the corresponding specific fragment ions for further efficient candidate selection in HPLC-HRMS analysis. In addition to the analytical aspect, a significant contribution to the novelty of the obtained results is made by the choice of plants growing in the subarctic climate of the European north of Russia as the object of research, a detailed study of the chemical composition of which has not been previously carried out.

## 2. Materials and Methods

### 2.1. Reagents and Materials

Acetonitrile (HPLC gradient grade), HPLC grade methanol, and methylene chloride were obtained from Khimmed (Moscow, Russia). ACS reagent grade formic acid (≥96%) and deuterated dimethyl sulfoxide (DMSO-d_6_, ≥99.8%) were purchased from Merck (Darmstadt, Germany).

A dedicated ACL reagent kit for photochemiluminescence (PCL) analysis was obtained from Analytik Jena AG (Jena, Germany), while a ready-to-use Folin–Ciocalteu reagent solution and an analytical standard of gallic acid (97.5–102.5%) were purchased from Sigma-Aldrich (Steinheim, Germany).

Ultrapure Type I water with a resistivity of 18.2 MΩ·cm obtained from a Milli-Q system (Millipore, Molsheim, France) was used for the preparation of the mobile phase for chromatographic separation.

*V. myrtíllus* and *V. uliginósum* plants were collected in the Primorsky district of the Arkhangelsk region of Russia (64° north latitude) in July 2022. The identification of the botanical material was carried out according to the herbarium of the Northern (Arctic) Federal University (voucher specimen numbers NARFU2022-VM and NARFU2022-VU).

### 2.2. Plant Material Extraction and Fractionation

The leaves and stems of *V. myrtíllus* and *V. uliginósum* were separated from the fruits, crushed, and then frozen for further research. The obtained samples (10 g) were subjected to ultrasonic treatment (35 kHz) 3 times for 20 min in an ultrasonic bath (Sapphire, Moscow, Russia) with a methanol–dichloromethane mixture (1:1, *v*/*v*). The obtained extract was fractionated by column chromatography using octadecyl silica Polygoprep 60–50 C_18_ (Macherey-Nagel, Duren, Germany) and four solvents (eluents) in order of decreasing polarity: water (1); 50% (*v*/*v*) aqueous methanol (2); methanol (3); and methanol–dichloromethane mixture, 1:1, *v*/*v* (4). As a result, four corresponding fractions (F1–F4) were obtained. The detailed conditions of the chromatographic separation are presented in [24,28].

### 2.3. In Vitro Antioxidant Activity and Total Polyphenols Content (TPC) Determination

Fractions were evaluated for the TPC using the Folin–Ciocalteu reagent as described previously [29]. All measurements were performed in a 96-well plate on a FlexA-200HT Microplate Reader (Hangzhou Allsheng Instruments Co., Hangzhou, China) at a wavelength of 750 nm in triplicate. The calibration curve was constructed using gallic acid as a standard. The methanolic solution (20 μL) of the dry sample with a concentration of 0.1–0.2 mg mL^−1^ was mixed with 100 μL of Folin–Ciocalteu reagent (10% aqueous solution) and incubated for 5 min at room temperature. Then, 80 μL of sodium bicarbonate solution (60 g L^−1^) was added, and the mixture was allowed to stand for 60 min.

The superoxide anion radical scavenging activity was measured via the use of 20 µL of sample solution in methanol (1 mg L^−1^) at a concentration of 50 mg L^−1^ and a commercially available ACL reagent kit via the PCL method on a Photochem analyzer (Analytik Jena AG, Jena, Germany) according to the known procedure [30] in three replicates. The calibration was performed immediately before the analysis using the Trolox standard solutions in methanol in the concentration range of 0.5–3 mg L^−1^.

### 2.4. Analytical Procedures

Two-dimensional HSQC (Heteronuclear Single Quantum Coherence) and HMBC (Heteronuclear Multiple Bond Correlation) NMR spectra were acquired on an AVANCE III 600 NMR spectrometer (Bruker, Ettlingen, Germany) using DMSO-d_6_ as the solvent. The specific experimental parameters of the acquisition and processing of the NMR data are presented in [25], as follows: for HSQC, a matrix consisting of 1024 × 256 points was obtained in 4 scans with a relaxation delay of 2 s; for HMBC, a matrix consisting of 2048 × 512 points was obtained in 8 scans with a relaxation delay of 2 s.

**Figure 1:**
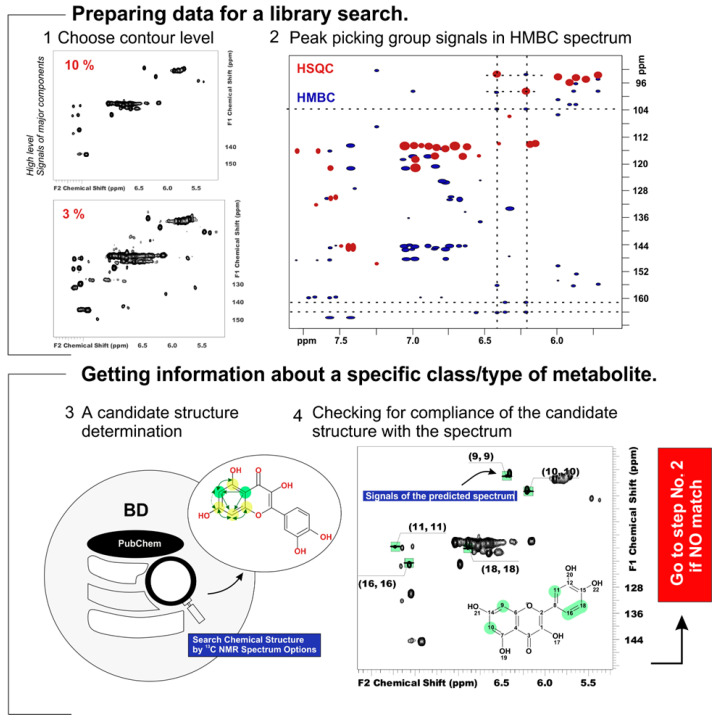
2D NMR analysis workflow.

The unknown screening and identification of organic compounds in plant extracts were carried out via ACD/Structure Elucidator expert system software v2019 (ACD/Labs, Toronto, ON, Canada) with an NMR spectral database and available literature data [3,9,16,17,20,22,31,32,33]. A detailed flowchart and an algorithm for identifying the chemical composition of the extract are presented in Figure 1.

The block diagram of complete or partial dereplication consists of four stages, which can be combined into two main groups. The first steps are necessary to prepare the data for library search (not including the steps of spectrum processing) and involve obtaining the contour plots at different levels of relative signal intensity and picking group signals on HSQC and HMBC spectra. The other steps focus on obtaining information about the type of the detected metabolite.

The dereplication of metabolites with a suitable structure or structural element according to established correlations was carried out via ACD/Structure Elucidator (ACD/Labs, Toronto, ON, Canada) computational expert system using integrated ChemSpider and/or PubChem chemical shift databases. The spectral search was carried out according to the data on the ^13^C chemical shifts with the following basic parameters: (1) rejection of structures with a compliance coefficient of >1 ppm; (2) the elemental composition of the analyzed substances was in the range of C (0–100), H (0–100), and O (0–20); (3) the HSQC data were used to check the chemical shifts of the attached hydrogens; and (4) information about the distance between atoms according to the HMBC or TOCSY spectra is obtained.

After detecting structures in the database that match the selected correlations, candidates were checked by the correspondence of their chemical shifts to the experimental spectrum. The candidate structure was considered true if the following conditions were met: (1) all target cross-peaks are present in the HSQC spectrum and (2) the maximum allowable error between the predicted chemical shifts and experimental data is <1 ppm for ^1^H and <5 ppm for ^13^C. When no candidate could be obtained during the spectral search or when none of the candidate structures came up again, step 2 was performed, eliminating suspicious interactions or adding new potential correlations. After identifying all the metabolites that have signals with a common contour level on the spectrum, then step 1 was repeated.

HPLC–HRMS analysis was carried out on an LC-30 Nexera (Shimadzu, Kyoto, Japan) liquid chromatography system with a UV-VIS detector coupled to an Orbitrap ID-X high-resolution mass spectrometer (Thermo Scientific, Waltham, MA, USA) with an orbital ion trap mass analyzer and an OptaMax NG ion source equipped with a heated electrospray ionization (HESI) probe. Chromatographic separation was achieved on a Nucleodur PFP column (Macherey-Nagel, Duren, Germany), 150 mm × 2 mm, 1.8 µm particle size, with a pentafluorophenyl reversed stationary phase. The mobile phase was a mixture of water (A) and acetonitrile (B), both of which contained 0.1% formic acid. The flow rate was 0.3 mL min^−1^, and the column temperature was 40 °C. For the analysis of the F2 and F3 fractions, the following gradient elution program was used: 0–3 min, 10% B; 3–40 min, linear ramp to 100% B; and 40–45 min, 100% B. In the case of fraction F4 containing the least polar analytes, the gradient elution program was modified as follows: 0–3 min 40% B; 3–25 min linear ramp to 100% B; and 25–30 min 100% B. The injection volume was 2.0 µL. A wavelength range of 220–800 nm, a spectral resolution of 4 nm, and an acquisition rate of 10 Hz were used in the spectrophotometric detection.

High-resolution mass spectrometry detection was carried out in negative (ESI−) ion electrospray ionization mode. The following ion source parameters were applied: spray voltage, 2.5 (ESI−) kV; sheath, auxiliary, and sweep gas (N_2_) flow rates of 50, 10, and 2 arb. units, respectively; ion transfer tube and vaporizer temperature of 325 and 350 °C, respectively; and S-lens RF level of 60%. Mass spectra were recorded in the *m*/*z* range of 100–1000; the mass analyzer resolving power was set to 120,000 (at *m*/*z* 200). Tandem (MS/MS) mass spectra were recorded in the data-dependent acquisition mode. The ions whose signal intensities exceeded a threshold value of 1.0 × 10^5^ cps were selected as precursor ions for MS/MS experiments. Higher-energy collision-induced dissociation (HCD) in stepped collision energy mode (20, 35, and 60%) with a mass analyzer resolving power of 30,000 was used in the tandem mass spectrometry analysis. To achieve the highest mass accuracy, an internal mass scale calibration (Easy-IC mode with fluoranthene as a standard) was used.

## 3. Results and Discussion

### 3.1. Polyphenol Content and Antioxidant Activity of the Obtained Fractions

In this work, to obtain a fraction enriched with polyphenolic compounds, ultrasonic extraction was performed with a mixture of methanol–dichloromethane (1:1) at room temperature. The extracts were then fractionated using column chromatography [28]. As already presented in a previous study [24], this approach allowed for the obtainment of a representative polyphenolic fraction free of carbohydrates, nonpolar triglycerides, and steroids. Although extraction was carried out for two closely related species, the total yield of extractives differed and was ~21% for *V. myrtíllus* versus 13% for *V. uliginósum* (Table 1).

Special attention should be given to the aqueous methanol fraction (F2). It is characterized not only by a high yield (8.7% for *V. myrtíllus* versus 4.6% for *V. uliginósum*) but also by the predominance of polyphenols (approximately half of the fraction weight), which have high antioxidant capacity (Table 1). Notably, the total content of polyphenols in both extracts was approximately the same (~425–440 mg eq. GA/g extract), but there was a significant difference in the antioxidant activity (AOA) in favor of the *V. uliginosum* extract. This clearly indicates differences in the chemical composition of the polyphenols contained in these extracts.

Although the methanolic fraction (F3) also contains polyphenols, their content is approximately 2–4 times lower, and the antioxidant activity is approximately 7 times lower than that of the F2 fraction. In this context, the aqueous methanol fraction was chosen to study the chemical composition of the polyphenolic compounds *V. myrtillus* and *V. uliginosum*.

A detailed analysis of the polyphenolic compounds in aqueous methanol extracts was carried out via a two-stage process. The basis is the non-targeted screening of the structures dominant in the extract by two-dimensional (2D) NMR spectroscopy (HSQC-HMBC combination) followed by a group search for the classes of metabolites detected by liquid chromatography–high-resolution mass spectrometry.

### 3.2. Annotating the Main Classes of Polyphenols by 2D NMR (Stage 1)

**Figure 2:**
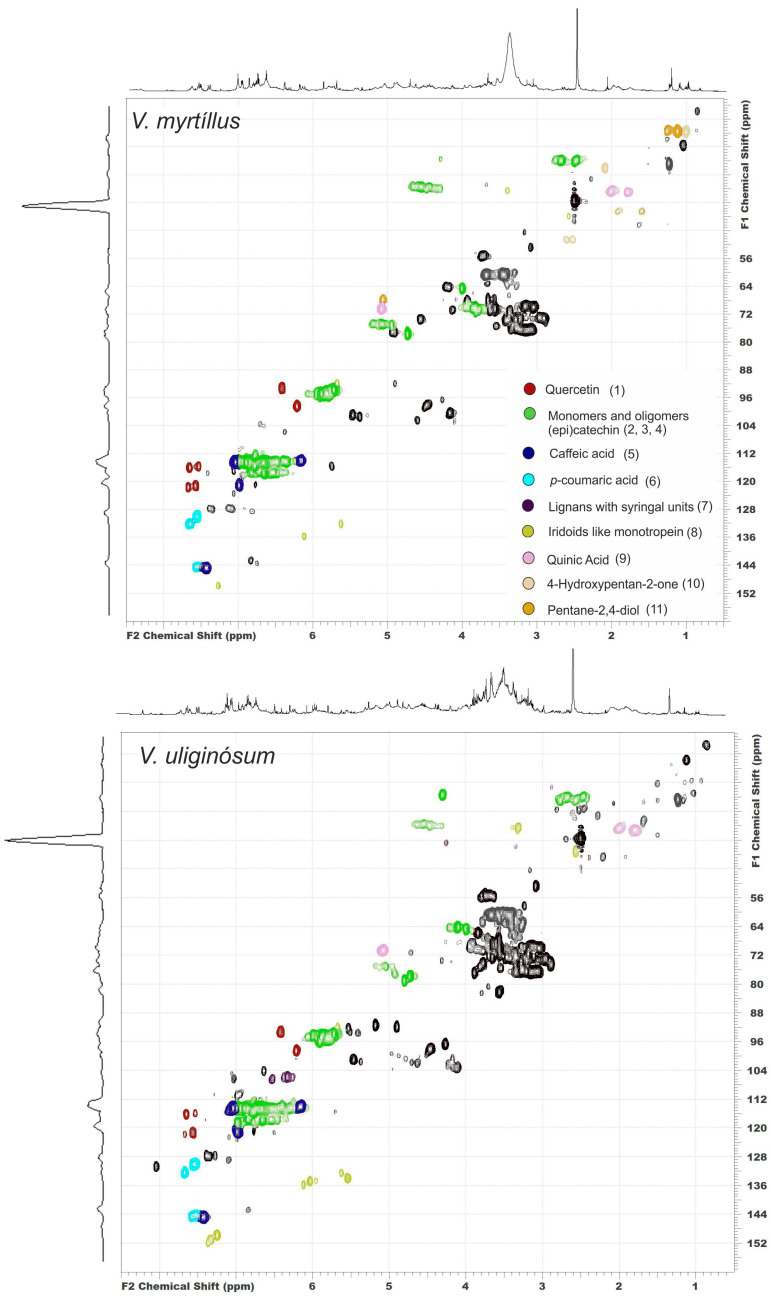
2D ^1^H-^13^C HSQC spectra of *V. myrtíllus* and *V. uliginósum* aqueous methanol extracts with the assignment of the main peaks.

**Figure 3:**
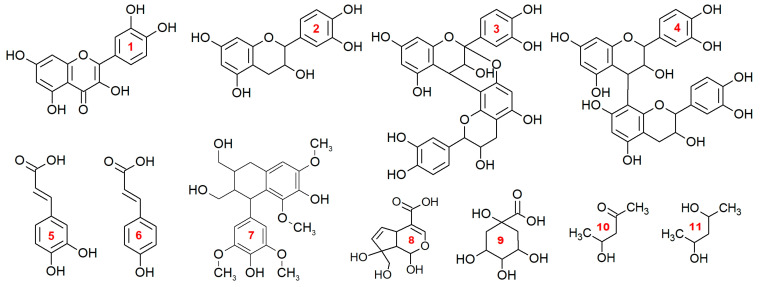
Structural formulas of the components of aqueous methanol extracts identified by 2D ^1^H-^13^C HSQC spectra: (**1**) quercetin; (**2**) (epi)catechin; (**3**) a type of proanthocyanidin; (**4**) B-type proanthocyanidin; (**5**) caffeic acid; (**6**) *p*-coumaric acid; (**7**) lyoniresinol; (**8**) iridoid (aglycon of the monotropein structure); (**9**) quinic acid; (**10**) 4-hydroxypentan-2-one; (**11**) pentane-2,4-diol.

HSQC NMR spectra with peak annotations obtained from the corresponding HMBC data (Figures S1 and S2) and the proposed structures of the detected compounds are shown in Figure 2 and Figure 3. Approximately 70 cross-peaks related to organic compounds of various classes are observed in each spectrum.

The analysis of correlations in the HMBC spectrum revealed that the main group of signals present in the aromatic region of the spectrum is related to the structures of two flavonoids: quercetin (93.31/6.41; 98.49/6.21; 116.1/7.65; and 121.2/7.57 ppm) and catechin (114.6/6.89; 117.6/6.66; 94.96/5.89; 93.78/5.72; 78.00/4.73; 64.72/4.00; 27.99/2.67; and 2.48 ppm). The latter is also present in the composition of proanthocyanidins (the target peak describing the C–C bond between two catechin units at 35.53/4.54 ppm). Notably, signals of two types of PAs (A and B) are largely present in the spectrum of the *V. uliginósum* extract, whereas only type B signals are observed in the spectrum of the *V. myrtíllus* extract. In both cases, in the immediate vicinity of the flavonoid signals, those corresponding to the structure of two types of hydroxycinnamic acids are also clearly traced: caffeic (114.2/7.03; 121.28/6.99; 114.2/6.16; and 144.95/7.42 ppm) and *p*-coumaric (130.07/7.52; 115.35/6.77; 114.85/7.43; and 114.04/6.15 ppm). In general, all the structures described above are components of most medicinal plants and are considered secondary metabolites in plants of the genus *Vaccinium* [3,9,20]. In addition, it was reported in the literature that the flavonol kaempferol is also a metabolite of both species [22]. Its structural formula differs from that of quercetin in the absence of one hydroxyl group in the aryl fragment. As a result, the signals of this fragment in the HSQC spectrum coincide with the signals of *p*-coumaric acid. In this case, its unambiguous identification in the HSQC spectrum of a metabolite mixture is difficult.

The signals at 149.9/7.24, 134.8/6.03, and 133.9/5.54 ppm are of the greatest interest. A detailed analysis of the long-range interactions of the described carbon peaks with protons at 36.5/3.32 and 43.1/2.56 ppm made it possible to assign them to the structure of an iridoid by the type of monotropein. Notably, three sets of signal data are present in the spectrum of the *V. uliginósum* extract, with a slight deviation in the chemical shift for the signals of the double bonds for the iridoid structure, which may indicate the presence of different substituents. The structures of iridoids are very specific but are widespread in plants of the genus *Vaccinium* [16,17,31]. In particular, the signals of the monotropein iridoid observed in the HSQC spectrum were previously detected during the identification of phenolic compounds from *V. vitis-idaea*, *V. myrtillus*, and *V. intermedium Ruthe* leaves [32] as well as *V. myrtillus* fruits [17].

Certain differences in the composition of the extracts can also be observed by analyzing the aliphatic region. Quinic acid signals (δ_C_/δ_H_ 70.64/5.08; 36.81/2.00; and 37.19/1.80 ppm) were observed in the spectra of both extracts. Along with the previously mentioned signals of caffeic and *p*-coumaric acids, the presence of chlorogenic and *p*-coumarylquinic acids in the extract may be indicated [21]. The spectrum of *V. myrtillus* extract also contains a set of peaks characteristic of 2,4-pentanediol. On the basis of data from the literature, it may be a part of a larger structure, for example, *p*-coumaryl-pentadiol-hexoside [32]. In addition, the signals with lower intensities at 105.8/6.33, 106.5/6.53, and 40.7/4.26 ppm are notable. Analysis of the correlation patterns observed in the HMBC spectrum suggested that they may be resonances of atoms in lignan structures, such as Lyoniside, which was previously found in *V. myrtillus* [33].

Thus, a preliminary analysis of the studied extracts via a combination of 2D NMR methods provided a detailed description of the chemical structure of the dominant components. After all possible dominant structures were identified, a detailed analysis of the chemical composition of the extracts was carried out via HPLC-HRMS in negative ion mode.

### 3.3. Molecular-Level Analysis of Polyphenolic Compounds by Liquid Chromatography–High-Resolution Mass Spectrometry (Stage 2)

A detailed analysis of aqueous methanol extracts (fraction F2) isolated from the stems and leaves of *V. myrtillus* and *V. uliginosum* was carried out by HPLC-ESI-HRMS in data-dependent tandem mass spectra acquisition (dd-MS2) mode. The obtained mass-spectrometry data were processed via an approach based on a group search for dominant polyphenolic structures detected by 2D NMR. To accomplish this, the chromatograms were reconstructed according to the exact *m*/*z* values of the product ion (only MS/MS spectra were used) corresponding to the aglycone structure ([M−H]^−^) of analytes belonging to a particular group. The detected chromatographic peaks indicate the presence of metabolites of the target classes in the extract; therefore, the selection of candidate compounds is greatly simplified. Further establishment of the proposed structure of the detected components was based on the elemental compositions of precursor ions, the study of tandem mass spectra, and the search for information in online databases and previously published works. This approach allowed the determination of the structures for more than 50 individual phenolic components that are dominant in extracts, including isomeric structures (Table 2).

**Figure 4:**
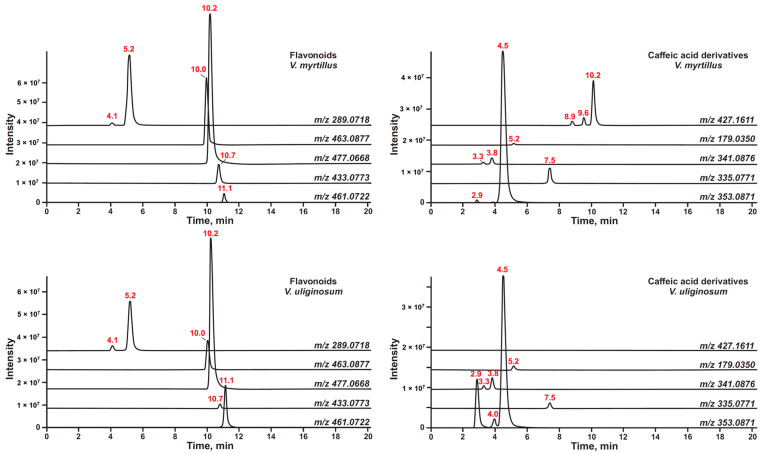
Accurate mass-based extracted ion chromatograms (XICs) of the detected flavonoids and caffeic acid derivatives in fraction F2.

Thus, the search for fragments with *m/z* values of 289.0718 and 301.0354 corresponding to the deprotonated molecules ([M−H]^−^) of (epi)catechin and quercetin, respectively, in the entire set of MS/MS spectra ensured the detection and tentative identification of at least eight metabolites belonging to the flavonoid class (Figure 4). Three of them are catechins: catechin (**2**), epicatechin (**3**), and the hexose-conjugated derivative (**1**). Among these, epicatechin is the most abundant.

Quercetin (**9**), in turn, can be found in extracts primarily in the form of sugar or uronic acid derivatives: 3-*O*-glucoside (isoquercitrin, **5**), 3-*O*-glucuronide (miquelianin, **6**), 3-*O*-arabinofuranoside (avicularin, **7**), and 3-*O*-disaccharide-rutinoside (rutin, **4**). The latter is found in small amounts only in the *V. myrtíllus* extract.

The polyphenolic fractions F2 isolated from *V. myrtillus* and *V. uliginosum* are generally characterized by a similar set of flavonoids. However, *V. myrtíllus* has a higher (2–5 times) content of various metabolites of this class (for example, epicatechin, avicularin, and others) than *V. uliginósum*.

In addition to various flavonoids, the search for metabolites by the product ion *m*/*z* 289.0718 made it possible to detect seven individual compounds (Table 2) from the class of proanthocyanidins—oligomers formed due to the polymerization of flavan-3-ol units (especially catechin and epicatechin) [34]. In terms of dimeric components, *V. myrtíllus* blueberry extract is characterized by the presence of only proanthocyanidins of type B (two isomers, **10**, **14**), which fully corresponds to the results of 2D-NMR. For *V. uliginósum*, proanthocyanidin A (**15**) is dominant, but both type B isomers are also present in significant amounts. Certain differences are also observed for trimeric structures: *V. myrtíllus* extract significantly exceeds (2–7 times) *V. uliginósum* in the content of two detectable types of B and A/B trimers (**11**, **12**). Moreover, up to three isomeric A/B trimer structures were found for the F2 fraction of *V. uliginosum* (**12**, **13**). As in the case of flavonoids, representatives of this class of compounds were previously described as metabolites in plants of the genus *Vaccinium* [4,9,21,35,36].

Besides proanthocyanidins, two other dimeric components related to flavonolignans were also detected by their characteristic product ion at *m*/*z* 289.0718. The main metabolite is Cinchonain I (**55**), which was previously found as a metabolite in plants of the genus *Vaccinium* [14]. The second component contains one fewer hydroxyl group (**56**). It is present only in the *V. myrtíllus* extract and has not been mentioned in literary sources before.

Aromatic acids and their derivatives constitute the next significant group of extractive substances. The search for these metabolites was also based on the results of preliminary screening by 2D NMR. The characteristic fragments were [C_9_H_7_O_4_]^−^ and [C_9_H_7_O_3_]^−^, corresponding to deprotonated molecules of caffeic and *p*-coumaric acids, respectively. This made it possible to detect a wide range of derivatives formed mainly via conjugation with sugar or quinic acid (Figure 3).

In the case of caffeic acid, this approach allowed for the detection of caffeoylhexosides (two isomers, **18**, **19**), caffeoylquinic (chlorogenic acid, three isomers, **17**, **20**, **21**), and caffeoylshikimic (**23**) acids, the contents of which in the studied fractions were close to each other. The exception is the isomer (*I*) of chlorogenic acid (**17**), the amount of which is two orders of magnitude greater in *V. uliginosum* than in *V. myrtillus*.

A similar pattern is observed for *p*-coumaric acid. Two isomers of *p*-coumaroylhexoside (**27, 29**) and four isomers conjugated with a quinic acid derivative (**28**, **30–32**) were found. The latter predominates in the *V. uliginosum* extract, which may be one of the main reasons for its higher antioxidant activity than the F2 fraction of *V. myrtillus*, which has a comparable total content of polyphenols.

Notably, *V. myrtíllus* is characterized by the presence of a wide range of metabolites belonging to the class of pentane-2,4-diol-glucoside derivatives with the aromatic acids presented above (eleven individual compounds, **24–26**, **33–40**), which could not be detected in *V. uliginósum*. The correctness of the identification of these components was confirmed by the elimination of fragments of monosaccharides (C_5_H_8_O_4_ and C_6_H_10_O_5_), as well as pentane-2,4–diol (C_5_H_10_O) (Figures S3 and S4, Supplementary Materials), a previously established structure by the 2D NMR method, in the high-resolution MS/MS spectra.

In addition to pentane-2,4-diol-glucosides, a targeted search for characteristic fragments of aromatic acids in the MS/MS spectra revealed the presence of another class of extractive substances: iridoid derivatives. The presence of these compounds has also been confirmed by both data from the literature [3,9,17,37] and the results of 2D NMR analysis (Figure 2). *V. myrtíllus* is characterized by the predominance of two iridoids in the isolated F2 fraction, isomers of *p*-coumaroyl monotropein (**45**, **48**), whereas *V. uliginósum* features a greater variety of component compositions. Thus, for *V. uliginosum*, three new representatives of the iridoid class were found, presumably identified as caffeoyl (**42**) and *p*-coumaroyl (**43**) hydroxydihydromonotropeins, as well as caffeoyl dihydromonotropein (**46**). The MS/MS spectra of these compounds demonstrate the elimination of caffeoyl (C_9_H_7_O_4_) or *p*-coumaroyl (C_9_H_7_O_3_) fragments, as well as fragments of dihydromonotropein with an additional hydroxyl group (C_16_H_21_O_11_), as shown in Figures S5 and S6 (Supplementary Materials).

Finally, a search was carried out for lignans, the aglycone structures of which were detected during 2D NMR screening. They are 5,5′-dimethoxy-isolariciresinol (lyoniresinol) and 5,5′-dimethoxy-secoisolariciresinol. When the MS/MS spectra were searched, their characteristic product ions were [C_22_H_27_O_8_]^−^ and [C_22_H_28_O_8_]^−^, respectively. The F2 fraction of *V. myrtíllus* contains only lyoniresinol conjugated with a pentose (Lyoniside, **51**). *V. uliginósum* are distinguished by the presence of four derivatives of lignans (two for each target aglycone structure), presumably having xylose (Ssioriside, **53**) or rhamnose (lyoniresinol-rhamnoside, **52** and Chaenomiside F, **54**) substituents in their structure, as confirmed by tandem mass spectra (Figure S7, Supplementary Materials).

### 3.4. Comparison of the Obtained Results with the Data from the Literatur

The use of the proposed approach to the group search for metabolites on the basis of a combination of complementary 2D NMR and HPLC–ESI–HRMS techniques made it possible to detect **56** individual compounds in the polyphenolic fractions of *V. myrtíllus* and *V. uliginósum*, 14 of which were discovered for the first time or were incorrectly identified earlier (marked in bold in Table 2). The latter group included the glycosides *p*-coumaroyl-pentanediol observed in the extract of *V. myrtíllus*. The presence of these metabolites in *V. myrtíllus* was unambiguously confirmed by the data presented in [32]. Importantly, several studies [9,31] have described some of the pentane-2,4-diol-glycosides listed in Table 2. However, the presence of a malonic acid fragment in the structure instead of a diol, which is characterized by the same molecular weight (104 Da), was mistakenly established. Clarification in identification, as well as the establishment of the presence of isomers previously unknown in the literature in extracts, allows for the discovery of at least eight new metabolites in the aerial part of *V. myrtillus*.

The group of first discovered compounds includes metabolites of two classes: iridoid derivatives and lignans. The presence of some iridoid derivatives has also been confirmed in the literature [3,9,17,37], but structures such as caffeoyl hydroxydihydromonotropein (**42**), *p*-coumaroyl hydroxydihydromonotropein (**43**), and caffeoyl dihydromonotropein (**46**) were identified in plants of the genus *Vaccinium* for the first time. Among the lignans, only Lyoniside (**51**) was previously found in the rhizomes and stems of *V. myrtillus* [33]. Other lignans, such as Ssioriside (**53**), lyoniresinol-rhamnoside (**52**), and Chaenomiside F (**54**), were first discovered as extractives in plants of the genus *Vaccinium*.

Importantly, the flavonoids presented in Table 2 include nine individual compounds (including eight detected on the basis of NMR screening), which were previously identified as metabolites extracted from plants of the genus *Vaccinium* [9,10,14,22,38]. The obtained results are an additional confirmation of the efficiency of the applied approach and the correctness of the assumed identification. Notably, Stanoeva et al. [3] did not observe monomeric (epi)catechin structures in bog bilberry leaf extracts. In addition to the abovementioned characteristic product ions, a search was also carried out for metabolites containing kaempferol ([M−H]^−^, *m*/*z* 285.0399). Despite the fact that their discrimination according to 2D NMR data in the studied extract was difficult, the presence of these structures is clearly indicated by a number of sources in the literature [20,21,22]. Indeed, one derivative (namely, kaempferol 3-*O*-glucuronide, **8**) was found in the isolated fractions, the content of which is comparable to that of other important representatives of this class. Like flavonoids, representatives of proanthocyanidins were previously described as metabolites in plants of the genus *Vaccinium* [4,9,21,35,36].

It should be noted that the results of this work do not consider phenolic compounds that are part of the insoluble components of plant raw materials [39], which is a limitation of this study.

## 4. Conclusions

This study aimed to analyze the chemical composition of soluble polyphenolic compounds in the stems and leaves of two plant species, *V. uliginosum* and *V. myrtillus*, using an improved two-stage analytical methodology based on 2D NMR and HPLC-HRMS techniques. The use of the proposed approach to the group search for metabolites made it possible to identify about 50 compounds related to various derivatives of flavonoids, cinnamic acids, iridoids, and lignans. A significant contribution to the novelty of the results obtained is made by the identification of a number of new compounds not previously identified in extracts of *V. myrtíllus* и *V. Uliginósum*, such as caffeoyl- and *p*-coumaroyl hydroxydihydromonotropein, as well as Ssioriside, lyoniresinol-rhamnoside and Chaenomiside F.

These findings may contribute to a better understanding of the pharmacological properties of *V. myrtillus* and *V. uliginosum* stems and leaves and the wider use of this undervalued raw material. Further application of the proposed approach, based on 2D NMR and HPLC-HRMS techniques, should also include the study of insoluble phenolic compounds. Insufficient knowledge of these compounds may seriously limit our understanding of valuable polyphenolic metabolites.

## Figures and Tables

**Table 1:** Characteristics of the studied fractions according to the total polyphenol content (TPC) and AOA according to the PCL assay.

		Yield, %	TPC, mg eq. GA/g	AOA, mg eq. Trolox/g
*V. myrtíllus*	F1	9.4 ± 0.2	- *	-
	F2	8.7 ± 0.2	425 ± 5	905 ± 9
	F3	1.5 ± 0.1	220 ± 10	273 ± 12
	F4	1.19 ± 0.01	-	-
*V. uliginósum*	F1	4.5 ± 0.2	-	-
	F2	4.6 ± 0.1	440 ± 3	1352 ± 14
	F3	2.75 ± 0.01	140 ± 8	182 ± 9
	F4	1.20 ± 0.01	-	-

*—below the detection limit.

**Table 2:** Major compounds in fraction F2 and their tentative identification by HPLC–ESI–HRMS.

N	t_R_, min	Formula	*m*/*z* [M−H]^−^	Δ*m*/*z*, ppm	Peak Area, ×10^8^		Putative Compound
					*V. myrtíllus*	*V. uliginósum*	
Flavonoids and their glycosides							
1	3.0	C_21_H_24_O_11_	451.1240	0.04	0.02	0.04	(epi)Catechin *O*-hexoside
2	4.1	C_15_H_14_O_6_	289.0718	0.16	0.10	0.16	Catechin
3	5.2	C_15_H_14_O_6_	289.0714	−1.11	6.03	3.12	Epicatechin
4	9.6	C_27_H_30_O_16_	609.1459	−0.28	0.03	-	Quercetin-3-*O*-rutinoside (Rutin)
5	10.0	C_21_H_20_O_12_	463.0877	−1.13	3.60	1.43	Quercetin 3-*O*-glucoside (Isoquercitrin)
6	10.2	C_21_H_18_O_13_	477.0668	−1.38	9.43	8.61	Quercetin 3-*O*-glucuronide (Miquelianin)
7	10.7	C_20_H_18_O_11_	433.0773	−0.70	1.08	0.20	Quercetin 3-*O*-arabinofuranoside (Avicularin)
8	11.1	C_21_H_18_O_12_	461.0722	−0.68	5.20	2.18	Kaempferol 3-*O*-glucuronide
9	13.7	C_15_H_10_O_7_	301.0354	−0.02	0.03	0.02	Quercetin
Proanthocyanidins							
10	4.1	C_30_H_26_O_12_	577.1346	−0.98	1.86	0.30	Proanthocyanidin B (I)
11	6.0	C_45_H_38_O_18_	865.1984	−0.20	0.68	0.10	Proanthocyanidin trimer B
12	6.4	C_45_H_38_O_18_	863.1826	−0.38	0.70	0.35	Proanthocyanidin trimer A/B (I)
13	7.0	C_45_H_38_O_18_	863.1827	−0.24	-	0.19	Proanthocyanidin trimer A/B (II)
14	8.8	C_30_H_26_O_12_	577.1349	−0.35	0.42	0.24	Proanthocyanidin B (II)
15	9.4	C_30_H_24_O_12_	575.1189	0.86	-	2.37	Proanthocyanidin A
16	10.0	C_45_H_38_O_18_	863.1824	−0.52	-	0.10	Proanthocyanidin trimer A/B (III)
Caffeic acid derivatives							
17	2.9	C_16_H_18_O_9_	353.0873	−1.31	0.01	1.21	Chlorogenic acid (I)
18	3.3	C_15_H_18_O_9_	341.0876	−0.64	0.04	0.05	Caffeoylhexoside (I)
19	3.8	C_15_H_18_O_9_	341.0876	−0.73	0.22	0.29	Caffeoylhexoside (II)
20	4.0	C_16_H_18_O_9_	353.0876	−0.80	0.04	0.29	Chlorogenic acid (II)
21	4.5	C_16_H_18_O_9_	353.0871	−2.09	9.24	6.32	Chlorogenic acid (III)
22	5.2	C_9_H_8_O_4_	179.0350	−0.13	0.05	0.11	Caffeic acid
23	7.5	C_16_H_16_O_8_	335.0771	−0.28	0.65	0.13	Caffeoylshikimic acid
24	8.9	C_20_H_28_O_10_	427.1611	0.31	0.11	-	Caffeoyl-pentanediol-hexoside (I) *
25	9.6	C_20_H_28_O_10_	427.1609	−0.05	0.23	-	Caffeoyl-pentanediol-hexoside (II) *
26	10.2	C_20_H_28_O_10_	427.1605	−0.55	1.77	-	Caffeoyl-pentanediol-hexoside (III) *
p-coumaric acids derivatives							
27	3.2	C_15_H_18_O_8_	325.0928	−0.17	0.16	0.13	*p*-coumaroylhexoside (I)
28	3.6	C_16_H_18_O_8_	337.0926	−0.89	-	1.24	*p*-coumaroylquinic Acid (I)
29	5.5	C_15_H_18_O_8_	325.0929	−0.08	0.08	0.03	*p*-coumaroylhexoside (II)
30	5.5	C_16_H_18_O_8_	337.0926	−0.80	0.26	1.12	*p*-coumaroylquinic Acid (II)
31	6.4	C_16_H_18_O_8_	337.0926	−0.53	0.25	1.11	*p*-coumaroylquinic Acid (III)
32	6.8	C_16_H_18_O_8_	337.0927	−0.44	0.83	2.54	*p*-coumaroylquinic Acid (IV)
33	9.5	C_26_H_38_O_14_	573.2192	0.52	0.03	-	*p*-coumaroyl-pentanediol-dihexoside (I) * (two separate monosaccharides in structure)
34	10.1	C_26_H_38_O_14_	573.2189	−0.01	0.16	-	*p*-coumaroyl-pentanediol-dihexoside (II) * (two separate monosaccharides in structure)
35	10.3	C_20_H_28_O_9_	411.1656	−1.06	1.89	-	*p*-coumaroyl-pentanediol-hexoside (I)
36	10.5	C_25_H_36_O_12_	543.2087	0.78	0.03	-	*p*-coumaroyl-pentanediol-hexoside-xyloside (I) * (two separate monosaccharides in structure)
37	10.6	C_20_H_28_O_9_	411.1655	−1.28	1.64	-	*p*-coumaroyl-pentanediol-hexoside (II)
38	10.8	C_26_H_38_O_14_	573.2189	0.09	0.06	-	*p*-coumaroyl-pentanediol-dihexoside (III) * (two separate monosaccharides in structure)
39	11.0	C_25_H_36_O_13_	543.2087	0.67	0.02	-	*p*-coumaroyl-pentanediol-hexoside-xyloside (II) * (two separate monosaccharides in structure)
40	11.5	C_20_H_28_O_9_	411.1654	−1.58	5.80	-	*p*-coumaroyl-pentanediol-hexoside (III)
Iridoids							
41	1.5	C_16_H_22_O_11_	389.1088	−0.36	0.13	0.80	Monotropein
42	4.6	C_25_H_30_O_15_	569.1512	0.31	-	0.03	Caffeoyl hydroxydihydromonotropein *
43	6.6	C_25_H_30_O_14_	553.1565	0.39	0.03	0.47	*p*-coumaroyl hydroxydihydromonotropein *
44	7.7	C_25_H_28_O_14_	551.1409	0.55	0.05	0.21	Caffeoyl monotropein
45	8.3	C_25_H_28_O_13_	535.1458	0.07	0.51	0.40	*p*-coumaroyl monotropein (I, Vaccinoside)
46	8.5	C_25_H_30_O_14_	553.1567	0.72	-	0.21	Caffeoyl dihydromonotropein *
47	8.9	C_25_H_30_O_13_	537.1615	0.48	0.01	0.27	*p*-coumaroyl-dihydromonotropein (I)
48	9.1	C_25_H_28_O_13_	535.1456	−0.50	1.52	0.95	p-coumaroyl monotropein (II, Vaccinoside)
49	9.7	C_25_H_30_O_13_	537.1613	−0.20	0.06	0.72	*p*-coumaroyl-dihydromonotropein (II)
Other							
50	1.4	C_7_H_12_O_6_	191.0560	−1.14	1.70	5.00	Quinic acid
51	9.0	C_27_H_36_O_12_	551.2134	0.41	0.18	0.41	Lyoniresinol-xyloside (Lyoniside)
52	9.4	C_28_H_38_O_12_	565.2291	0.03	-	0.40	Lyoniresinol-rhamnoside *
53	10.3	C_27_H_38_O_12_	553.2290	0.47	-	0.09	5,5′-dimethoxy-secoisolariciresinol-xyloside (Ssioriside) *
54	10.7	C_28_H_40_O_12_	567.2445	−0.34	-	0.13	5,5′-dimethoxy-secoisolariciresinol-rhamnoside (Chaenomiside F) *
55	11.6	C_24_H_20_O_9_	451.1032	−0.07	0.65	0.13	Cinchonain I
56	12.7	C_24_H_20_O_8_	435.1085	−0.05	0.12	-	Cinchonain I derivate *

* First discovered or previously incorrectly identified.

## Data Availability

The data presented in this study are available in the article and the Supplementary Materials.

## Supplementary Materials

The following supporting information can be downloaded at: https://www.mdpi.com/article/10.3390/antiox13111409/s1, Figure S1: 2D ^1^H-^13^C HMBC spectrum of the aqueous methanol extract of *V. myrtillus* leaves and stems; Figure S2: 2D ^1^H-^13^C HMBC spectrum of the aqueous methanol extract of *V. uliginosum* leaves and stems; Figure S3: tandem mass spectrum of the caffeoyl-pentanediol-hexoside isomer (III); Figure S4: tandem mass spectrum of the *p*-coumaroyl-pentanediol-hexoside-xyloside isomer (II) with two separate monosaccharides in structure; Figure S5: tandem mass spectrum of the Caffeoyl hydroxydihydromonotropein; Figure S6: tandem mass spectrum of the *p*-coumaroyl hydroxydihydromonotropein; Figure S7: tandem mass spectrum of the lyoniresinol-rhamnoside.
