# Supplementary material for: Polyphenolic Antioxidants in Bilberry Stems and Leaves: A Non-Targeted Analysis by Two-Dimensional NMR Spectroscopy and Liquid Chromatography–High-Resolution Mass Spectrometry
Source: Antioxidants (Basel). 2024 Nov 17;13(11):1409. doi: 10.3390/antiox13111409 (PMC11591115; doi:10.3390/antiox13111409)
Supplement: Supplementary file 1 [file antioxidants-13-01409-s001.zip › antioxidants-3281306-supplementary.pdf]

Supplementary material

Polyphenolic antioxidants in bilberry stems and leaves: non-targeted analysis by two-dimensional NMR spectroscopy and liquid chromatography – high-resolution mass spectrometry

Anna V. Faleva*, Nikolay V. Ulyanovskii, Alexandra A. Onuchina and Dmitry S. Kosyakov

Laboratory of Natural Compounds Chemistry and Bioanalytics, Core Facility Center "Arktika", M.V. Lomonosov Northern (Arctic) Federal University, Northern Dvina Emb. 17, 163002, Arkhangelsk, Russia;
n.ulyanovsky@narfu.ru (N.V.U.); a.onuchina@narfu.ru (A.A.O.); d.kosyakov@narfu.ru (D.S.K.)
*Correspondence: a.bezumova@narfu.ru (A.V.F.)

Figure S1: 2D ^{1}H-^{13}C HMBC spectrum of aqueous methanol extract of *V. myrtillus* leaves and stems
Figure S2: 2D ^{1}H-^{13}C HMBC spectrum of aqueous methanol extract of *V. uliginosum* leaves and stems
Figure S3: Tandem mass spectrum of the Caffeoyl-pentanediol-hexoside isomer (III) (retention time 10.2 min, precursor ion 427.1605) observed in negative ion detection mode;
Figure S4: Tandem mass spectrum of the *p*-Coumaroyl-pentanediol-hexoside-xyloside isomer (II) with two separate monosaccharides in structure (retention time 11.0 min, precursor ion 543.2087) observed in negative ion detection mode;
Figure S5: Tandem mass spectrum of the Caffeoyl hydroxydihydromonotropein (retention time 4.6 min, precursor ion 569.1512) observed in negative ion detection mode;
Figure S6: Tandem mass spectrum of the *p*-coumaroyl hydroxydihydromonotropein (retention time 6.6 min, precursor ion 553.1565) observed in negative ion detection mode;
Figure S7: Tandem mass spectrum of the Lyoniresinol-rhamnoside (retention time 9.4 min, precursor ion 565.2291) observed in negative ion detection mode.

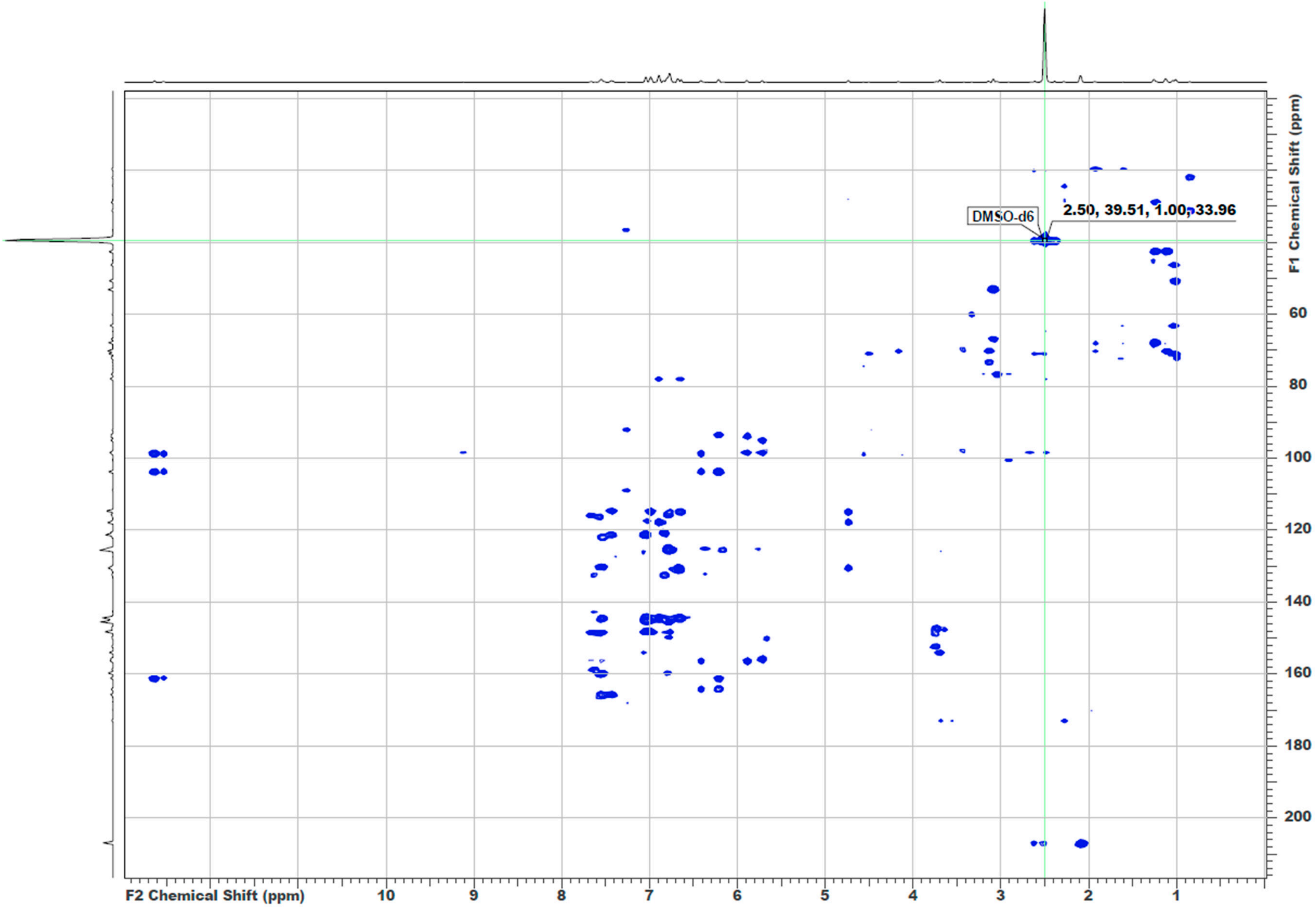

Figure S1: 2D ^{1}H-^{13}C HMBC spectrum of aqueous methanol extract of *V. myrtillus* leaves and stems

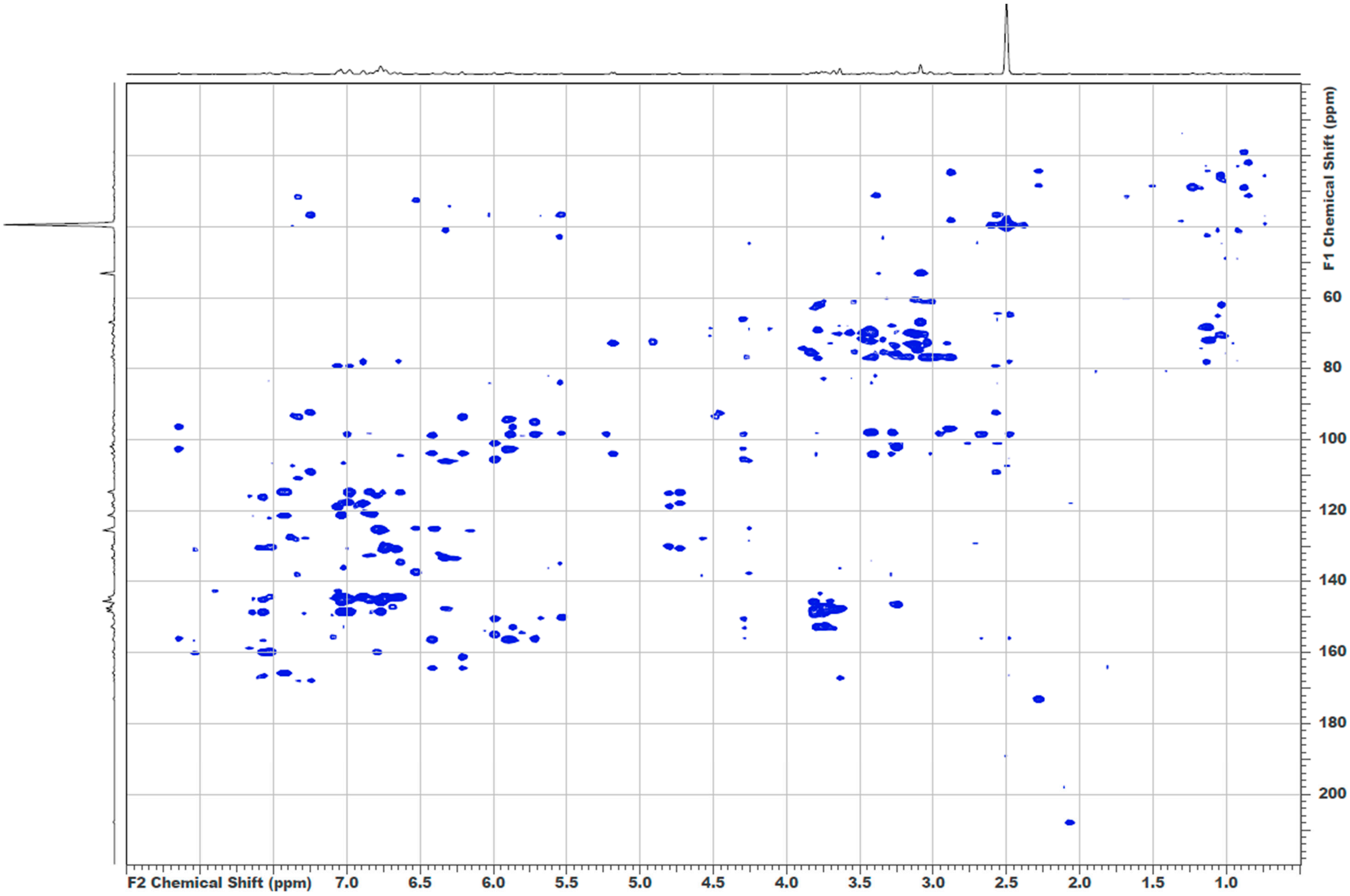

Figure S2: 2D ^{1}H-^{13}C HMBC spectrum of aqueous methanol extract of *V. uliginosum* leaves and stems

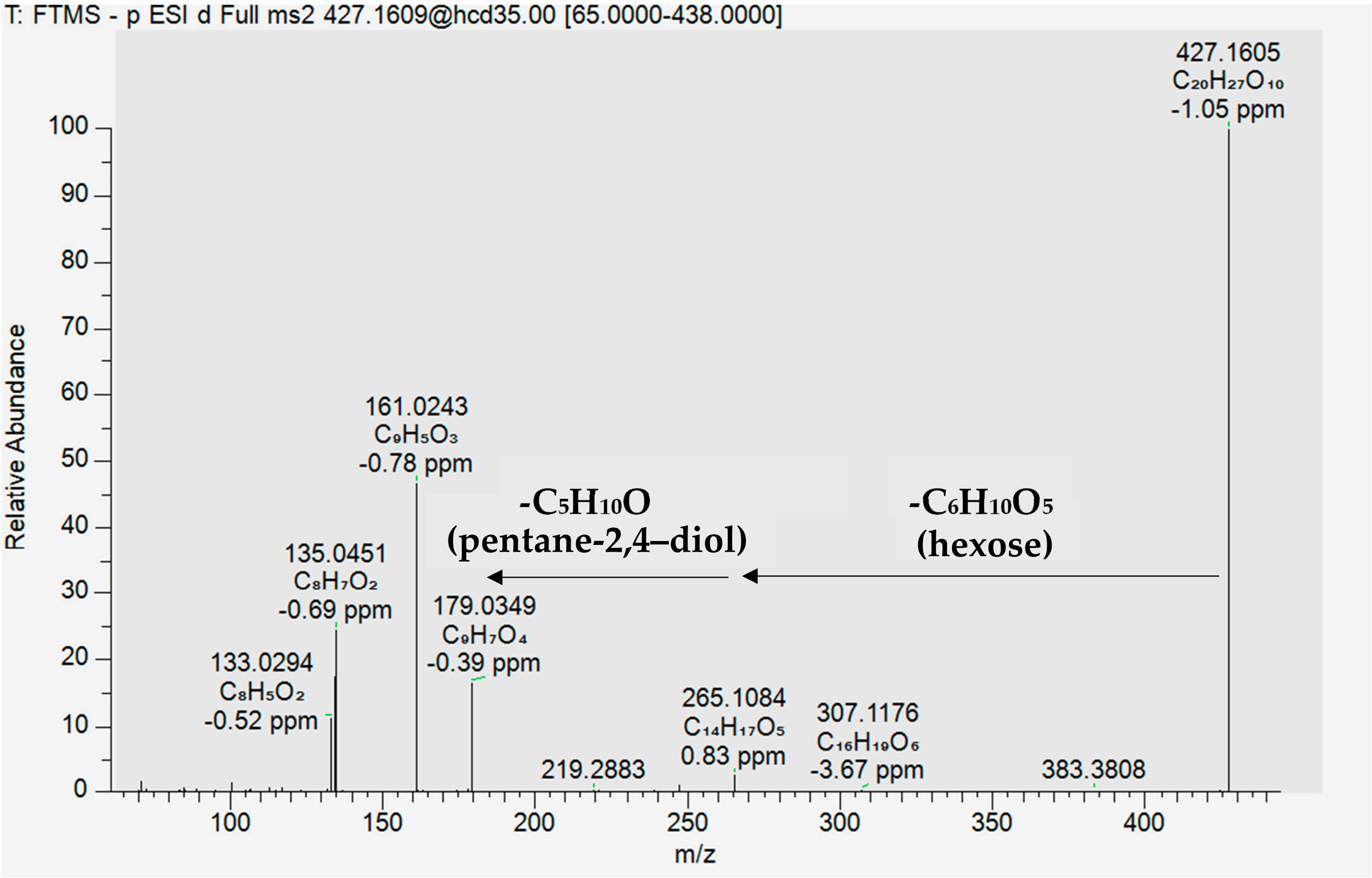

Figure S3: Tandem mass spectrum of the Caffeoyl-pentanediol-hexoside isomer (III) (retention time 10.2 min, precursor ion 427.1605) observed in negative ion detection mode

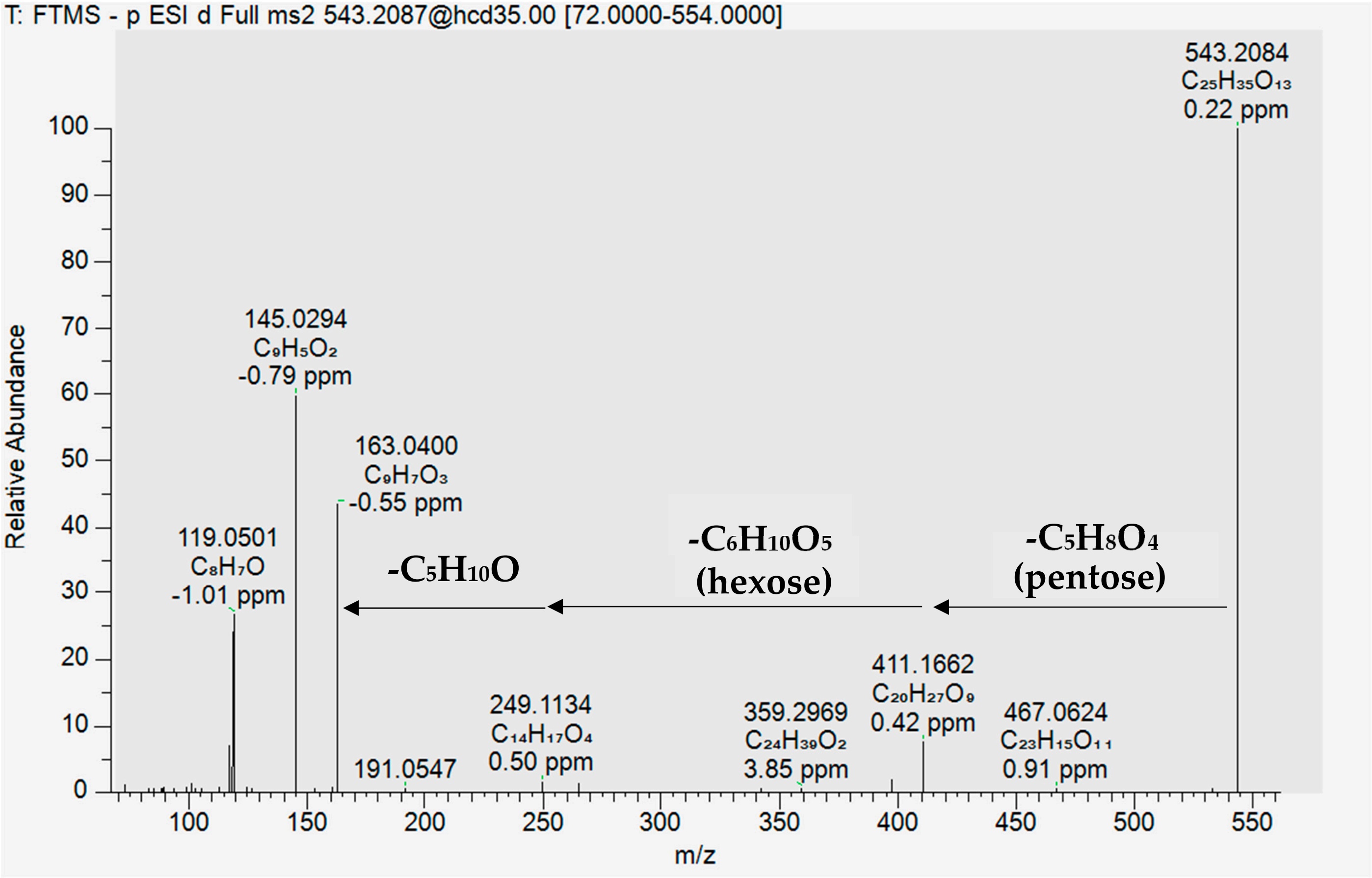

Figure S4: Tandem mass spectrum of the p-Coumaroyl-pentanediol-hexoside-xyloside isomer (II) with two separate monosaccharides in structure (retention time 11.0 min, precursor ion 543.2087) observed in negative ion detection mode

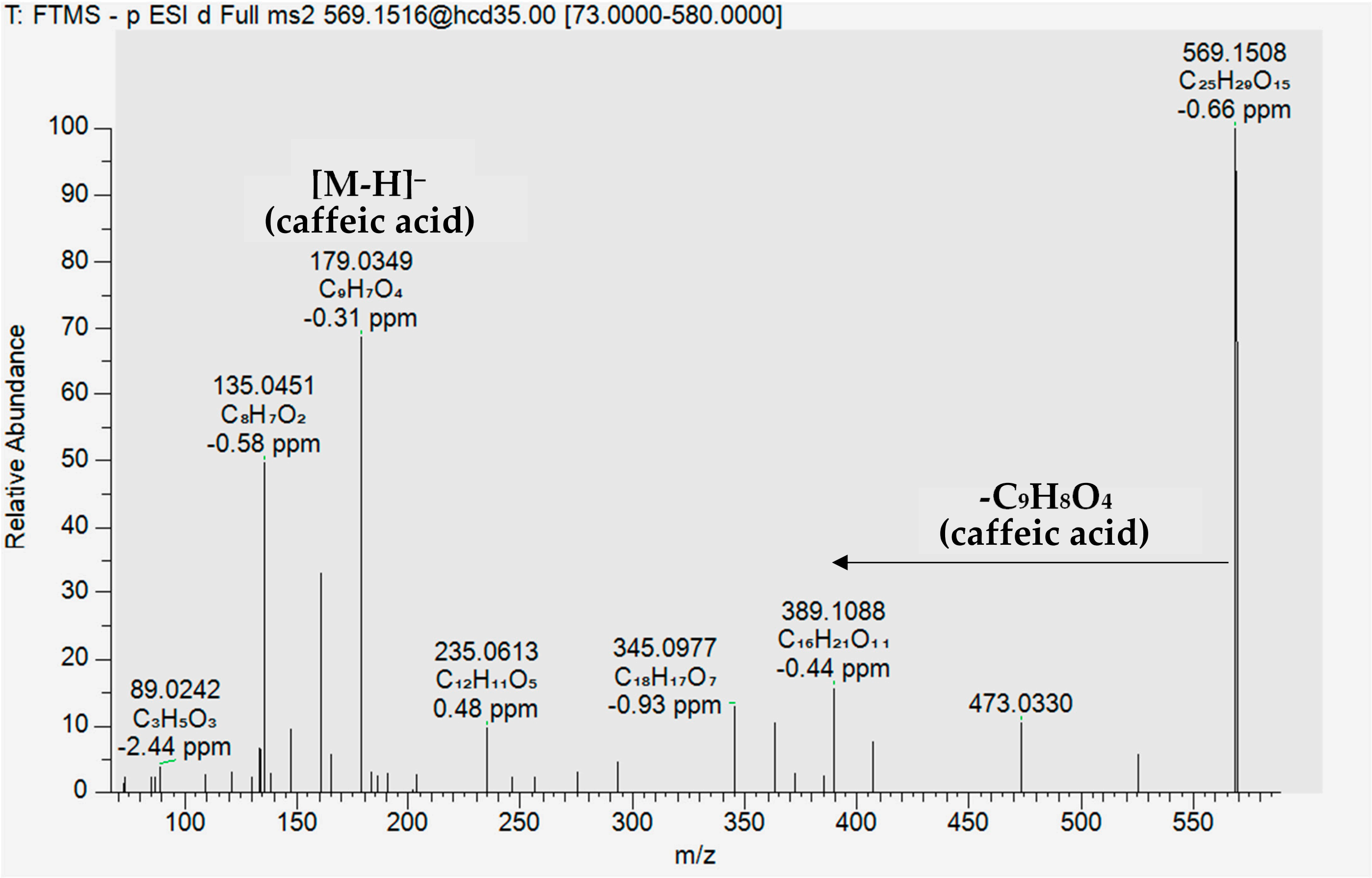

Figure S5: Tandem mass spectrum of the Caffeoyl hydroxydihydromonotropein (retention time 4.6 min, precursor ion 569.1512) observed in negative ion detection mode

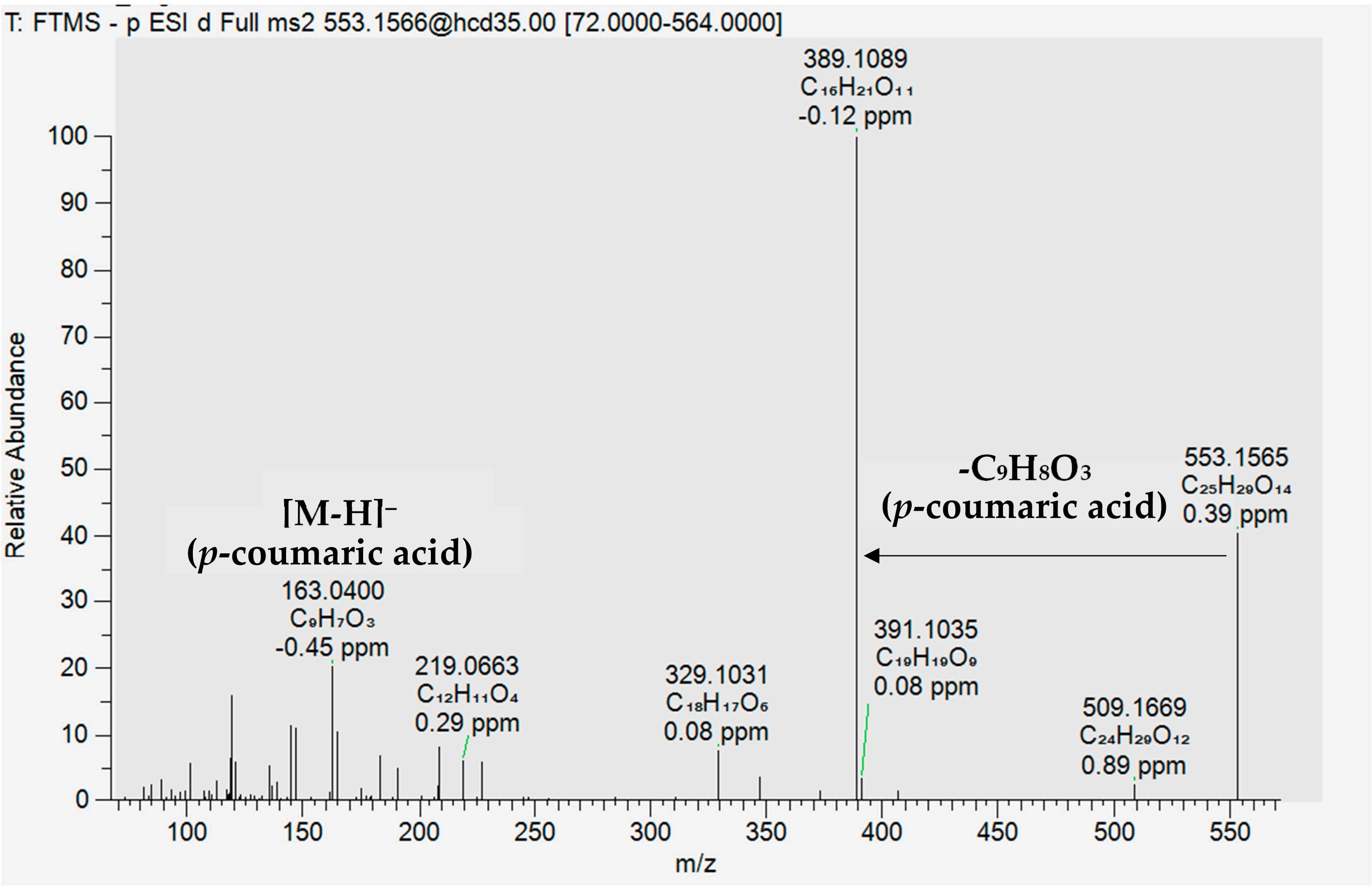

Figure S6: Tandem mass spectrum of the *p*-coumaroyl hydroxydihydromonotropein (retention time 6.6 min, precursor ion 553.1565) observed in negative ion detection mode

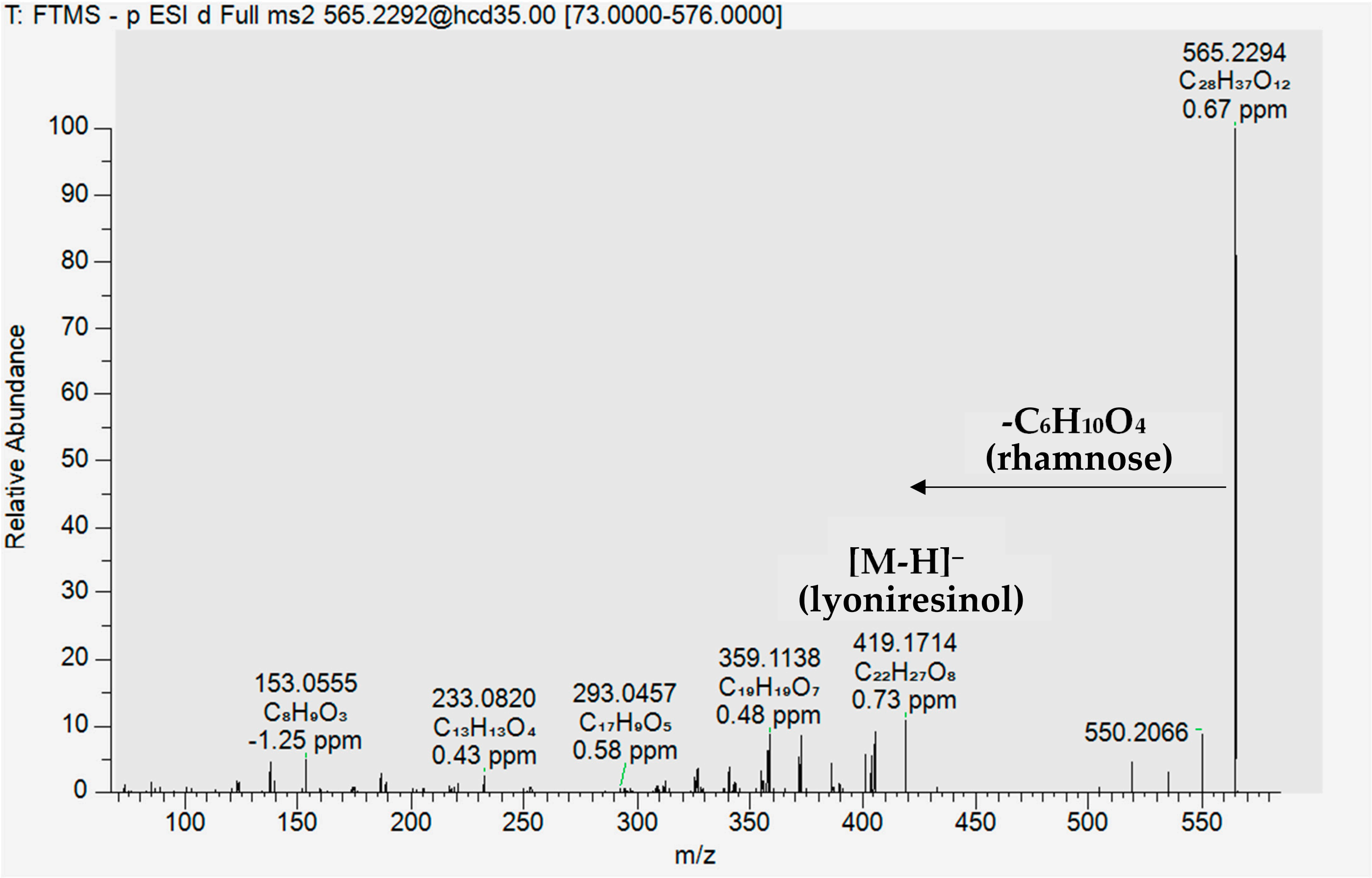

Figure S7: Tandem mass spectrum of the Lyoniresinol-rhamnoside (retention time 9.4 min, precursor ion 565.2291) observed in negative ion detection mode
